# Acute Respiratory Failure in Autoimmune Rheumatic Diseases: A Review

**DOI:** 10.3390/jcm13103008

**Published:** 2024-05-20

**Authors:** Sofia Poli, Francesca Sciorio, Giorgio Piacentini, Angelo Pietrobelli, Luca Pecoraro, Sara Pieropan

**Affiliations:** Pediatric Clinic, Department of Surgical Sciences, Dentistry, Pediatrics and Gynecology, University of Verona, 37126 Verona, Italy

**Keywords:** rheumatic diseases, respiratory failure, intensive care unit (ICU)

## Abstract

This review examines respiratory complications in autoimmune rheumatic diseases within intensive care units (ICUs). The respiratory system, primarily affected in diseases like rheumatoid arthritis, systemic lupus erythematosus, and scleroderma, often leads to respiratory failure. Common manifestations include alveolar hemorrhage, interstitial fibrosis, and acute respiratory distress syndrome. Early recognition and treatment of non-malignant conditions are crucial to prevent rapid disease progression, with ICU mortality rates ranging from 30% to 60%. Delayed immunosuppressive or antimicrobial therapy may result in organ system failure. Collaboration with rheumatic specialists is vital for accurate diagnosis and immediate intervention. Mortality rates for rheumatic diseases in the ICU surpass those of other conditions, underscoring the need for specialized care and proactive management. The review emphasizes comprehensive assessments, distinguishing disease-related complications from underlying issues, and the importance of vigilant monitoring to enhance patient outcomes.

## 1. Introduction

In recent years, interest has focused on intensive care manifestations of rheumatologic disorders, especially on pulmonary involvement. The respiratory system, which may rarely be the initial site of manifestation of a rheumatologic disorder [1], is the organ most commonly involved in the ICU [2] (e.g., diffuse alveolar hemorrhage, shrinking lung syndrome), followed by the renal, cardiac, gastrointestinal, and nervous systems. Recent trials have demonstrated that respiratory failure is a more common cause of ICU admission than infections [3,4,5]. The main purpose of this review is to evaluate in the literature the most frequent causes of respiratory failure in patients admitted to ICUs affected by common autoimmune rheumatic diseases.

## 2. Materials and Methods

A comprehensive literature search was conducted across multiple databases, including PubMed, the Journal of Rheumatology, the New England Journal of Medicine, the Journal of the American Medical Association (JAMA), and ResearchGate to identify relevant articles exploring the association between autoimmune rheumatic diseases and acute respiratory failure, along with underlying immunological mechanisms, diagnostic criteria, management strategies, and outcomes. Inclusion criteria comprised clinical studies and review articles addressing these topics, while exclusion criteria included non-English articles, duplicates, and those not directly pertinent to the review’s scope. Data extraction focused on key study elements such as design, patient characteristics, interventions, outcomes, and conclusions, synthesized to provide a comprehensive overview. Quality assessment considered study design, risk of bias, and methodological rigor. The reporting adhered to the Preferred Reporting Items for Systematic Reviews and Meta-Analyses (PRISMA) guidelines.

## 3. Rheumatic Diseases in the ICU

Frequently, systemic rheumatic diseases may have complications and medical emergencies that create a diagnostic problem due to their protean manifestations. Hence, identification of the conditions that pose a potential danger or risk to life in patients diagnosed with connective tissue diseases and vasculitis is of outmost importance in the ICU in order to mitigate the swift progression of morbidity and mortality [6]. Patients with systemic rheumatic diseases may be admitted to critical care settings for exacerbation of the underlying rheumatic condition (25% to 35%), for development of new manifestations, for infections (50%) caused by immunosuppression, or for adverse effects of medications [7]. Rheumatoid arthritis is the predominant rheumatic disorder observed among ICU admissions, followed by systemic lupus erythematosus and scleroderma. These three conditions constitute up to 75% of rheumatic cases admitted to intensive care units. While a subset of patients may present with acute illnesses unrelated to their underlying rheumatologic condition, it is noteworthy that the presence of such rheumatic disorders can exert a notable influence on the progression and outcome of these acute medical conditions [7]. The major respiratory manifestations encountered are alveolar hemorrhage (AH), chronic interstitial pulmonary fibrosis, acute respiratory distress syndrome (ARDS), thromboembolism of lung arteries; less common are shrinking lung syndrome, lupus pneumonia, muscular pulmonary disease, pleural effusions, tracheobronchial collapse, and subglottic obstruction [7]. A comprehensive approach is warranted in the ICU to promptly ascertain the diagnosis, whether it pertains to exacerbations of the rheumatologic disorder or infectious origins. Delaying administration of suitable immunosuppressive or antimicrobial treatment could result in multiple organ system failures and contribute to an adverse prognosis.

### Prognosis

As shown in Table 1, few studies have examined the overall prognosis [3,4,8,9,10,11] and the prognostic factors [6,8]. Mortality rates ranging from 30% in the ICU to 64% in the hospital [3] have been reported. Godeau investigated the short- and long-term results among 181 patients, discovering that, in addition to elevated Simplified Acute Physiology Score II (SAPS II) scores, pre-existing poor health status and corticosteroids were linked to unfavorable outcomes in the ICU [8]. Thong et al. identified that the prolonged duration of rheumatic disease before admission to the intensive care unit, coupled with the administration of high doses of corticosteroids or immunosuppressive agents, was associated with an unfavorable prognosis [3]. These studies consistently indicate that ICU mortality rates are higher in patients admitted for infections [3,8] compared to those admitted for flare-ups of rheumatologic conditions [4]. According to Pourrat et al., admission to the ICU for infection is linked to a mortality rate that may surpass 50%. The overall ICU mortality rate among patients with systemic rheumatic diseases ranges from 30% to 60% [4,6], significantly exceeding the projected mortality rate calculated using the Acute Physiology and Chronic Health Evaluation (APACHE) II or Simplified Acute Physiology Score (SAPS) II scores [3,8]. These mortality rates exceed those observed in patients lacking rheumatic diseases upon admission to ICU [12]. Ngueyn-Oghalai et al. documented 16 fatalities, with infections contributing to five deaths, renal failure to two, disseminated intravascular coagulation to two, severe trauma to one, and pulmonary embolism to one [12]. Among the cohort of 33 patients, in Pourratt’s study, 10 fatalities were recorded: six attributed to infection, three to disease exacerbation, and one to an unspecified cause [4].

## 4. Manifestations of Acute Respiratory Failure

### 4.1. Alveolar Hemorrhage (AH)

Alveolar hemorrhage (AH) predominantly affects systemic lupus erythematosus (SLE) and systemic vasculitis patients, less commonly in dermatomyositis, Goodpasture’s syndrome, and Wegener’s granulomatosis [7,13]. Vasculitis-induced small blood vessel inflammation causes diffuse lung air sac bleeding and tissue swelling, damaging arteries, capillaries, and veins. Symptoms include breathing difficulty, abnormal lung imaging (100% of cases), and high body temperature (82%), with hemoptysis in only about half [14,15]. Reduced hematocrit levels (75–100% of cases) are typical, often leading to misdiagnoses like infectious pneumonia or lupus pneumonitis [16]. The list of potential diagnoses (as provided in Table 2) encompasses infectious pneumonia, acute lupus pneumonitis, conditions characterized by capillaritis on histopathological examination (including vasculitis of unknown cause and vasculitis secondary to other conditions), and conditions marked by non-specific bleeding [17]. AH in SLE has a mortality rate of 70–90%, with manifestations ranging from mild to life-threatening [18,19]. Capillaritis onset in SLE correlates with AH, resembling lupus-related microangiopathy. In cases lacking hemoptysis, declining red cell indices alongside pulmonary infiltrate and progressive glomerulonephritis complicate AH identification [14,15]. Commonly, pulmonary and renal involvement may present together, known as pulmonary-renal syndromes, with differential diagnoses including anti-glomerular basement membrane (anti-GBM) disease, antineutrophil cytoplasmic autoantibodies (ANCA)-associated vasculitis, and systemic lupus erythematosus (SLE), as outlined in Table 3. Reduced serum complement levels typically indicate systemic lupus or postinfectious glomerulonephritis, although right-sided endocarditis patients may sporadically exhibit pulmonary infiltrates, diminished complement levels, and glomerulonephritis [20]. Normocomplementemic syndrome often stems from systemic vasculitis or anti-GBM disease; therefore, assessing ANCA and anti-GBM antibody levels is informative [21]. Kidney biopsies, immunofluorescence, and discerning circulating autoantibodies contribute significantly to diagnosis. Detecting circulating anti-GBM antibodies confirms the diagnosis and a subset of patients with the anti-GBM disease may also exhibit positive serology for p- or c-ANCA [20,21]. In contrast, only 8% to 10% of patients primarily diagnosed with ANCA-associated diseases like Wegener’s granulomatosis also exhibit the presence of anti-GBM antibodies [20]. Notably, serum complement levels typically remain normal (Table 3). While renal biopsy is recommended for both anti-GBM disease and ANCA-related vasculitis, in the case of anti-GBM disease, therapy initiation should not be delayed pending histologic confirmation [22,23]. The absence of characteristic immunofluorescence patterns, such as linear IgG deposition in Goodpasture syndrome or irregular Ig and complement deposition in SLE, is a guide for excluding other conditions [24]. Alveolar hemorrhage warrants consideration in patients with puzzling diffuse infiltration throughout the lungs or when diffuse pulmonary conditions coexist with connective tissue disorders, bone marrow transplants, chemotherapy, and particularly in instances of recent kidney function decline [23,24]. This scenario may precipitate respiratory failure, necessitating mechanical ventilation for over half of the affected individuals in the majority of reported datasets. Invasive hemodynamic monitoring is required to avoid exacerbation of pulmonary edema/hemorrhage and acute respiratory distress syndrome, which would reduce lung compliance. A small-scale study of seven patients with severe vasculitis admitted to the ICU demonstrated that two developed tension pneumothorax when protective ventilation measures were not applied [25,26]. For uncertain diagnoses, bronchoscopy with broncho-alveolar lavage may be necessary, revealing macrophages laden with hemosiderin or pigment alongside negative bacterial and fungal cultures [15]. Bronchoscopy is recommended as validated tool for diagnosis; in addition, it rules out infections [13]. Initiating prompt treatment with high-dose glucocorticosteroids, cyclophosphamide, and/or plasmapheresis could be crucial for patient survival. The mortality rate among patients with alveolar hemorrhage is substantial, with most studies reporting rates ranging from 40% to over 90% [14,27].

### 4.2. Interstitial Lung Disease (ILD)

Interstitial lung diseases are linked to different rheumatic conditions and encompass a range of histopathological types, such as non-specific interstitial pneumonia (NSIP), bronchiolitis obliterans organizing pneumonia (BOOP, also known as cryptogenic organizing pneumonia), apical fibrosis, diffuse alveolar damage (DAD), and lymphocytic interstitial pneumonia (LIP). The histopathological and radiological profiles of interstitial lung diseases linked to collagen exhibit similarities to those of their idiopathic counterparts [28].

In rheumatologic disorders, airway complications may manifest without an identifiable cause or as a result of therapy (like D-penicillamine or intramuscular gold compounds). These complications can escalate swiftly and, if left untreated, may lead to fatal outcomes [29]. Pulmonary involvement in systemic sclerosis is undoubtedly more widespread and critical than in other types of collagen diseases. It accounts for significant lifetime morbidity and is the leading cause of death. The most common pulmonary manifestation is interstitial fibrosis with histologic features of non-specific or, more commonly, usual interstitial pneumonia; pulmonary fibrosis tends to manifest with greater severity in patients exhibiting diffuse skin forms. Patients with ILD commonly have a rapid decline in pulmonary function that occurs in conjunction with progressive skin disease. Dyspnea on exertion without chest pain is the most common presenting symptom, with a dry cough being a late manifestation of ILD. Chest radiographs may have limited sensitivity in detecting lung disease, particularly in cases of diffuse interstitial lung disease (ILD). However, when abnormalities are present, they may manifest as bilateral reticulonodular changes in the lower lobes of the lung parenchyma, which can suggest fibrosis or interstitial lung involvement [30]. Systemic inflammatory disorders such as polymyositis (PM) and dermatomyositis (DM) can be associated with diffuse interstitial lung disease. The frequency of ILD in PM and DM varies widely, reported to be between 5% and 30%, depending on the diagnostic method used and the population studied. Most patients with pulmonary symptoms have what appears to be persistent community-acquired pneumonia refractory to antibiotic therapy.

**Figure 1:**
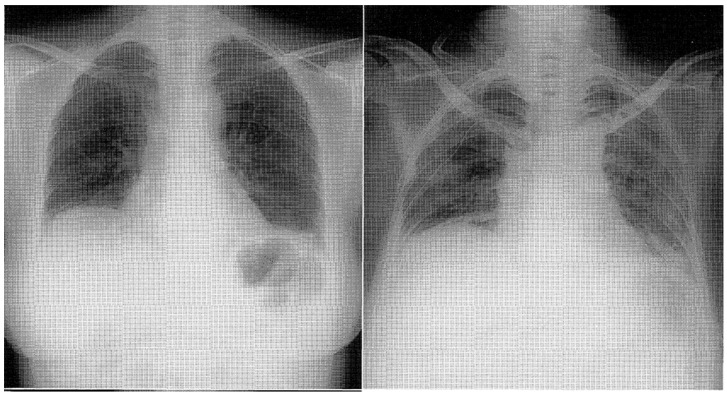
Chest X-ray displaying reticulonodular shadows in both lower lung fields [37].

The study by W.W. Douglas et al. highlighted that among patients with polymyositis (PM) and dermatomyositis (DM), the most frequently associated form of interstitial lung disease (ILD) was nonspecific interstitial pneumonia (NSIP) [31]. NSIP is commonly associated with the presence of specific autoantibodies, particularly anti-Jo1 and other anti-aminoacyl-tRNA synthetase autoantibodies. This association is part of a clinical syndrome known as the “antisynthetase syndrome”. Fulminant Pneumocystis Carinii pneumonia has been reported in four patients with human immunodeficiency virus-negative dermatomyositis who were lymphopenic before initiation of corticosteroids [32]; Nocardia pleural empyema complicated the disease in a Jo-1 positive polymyositis patient during treatment with intravenous immunoglobulin and corticosteroids [33]. Non-specific interstitial pneumonia is frequently encountered with varying proportions, especially in progressive forms compared to other systemic rheumatic diseases, particularly rheumatoid arthritis (RA) and systemic sclerosis. However, it may dominate the clinical picture in some patients [34]. ILD in patients with RA has a male predominance frequently towards 60 years of age. Most patients have usual interstitial pneumonia, and only a small percentage have histologic findings of non-specific pneumonia [28]. Advanced interstitial lung disease (ILD) can lead to significant cardio-pulmonary and respiratory failure, resulting in a poor prognosis for affected individuals. The prognosis of advanced ILD is generally poor, with a 5-year survival rate of around 50% [29]. Furthermore, the association of fibrosing alveolitis with antiphospholipid syndrome (APS) is rare and has been reported in only two cases [35]. High-resolution computed tomography (HRCT) has emerged as a valuable tool for detecting and characterizing interstitial lung diseases (ILDs), including those associated with collagen diseases. Compared to chest radiography and conventional CT, HRCT offers superior spatial resolution and image quality, allowing for detailed evaluation of lung parenchymal abnormalities. There is evidence that the pattern of abnormality at high-resolution CT reflects the relative proportions of fibrosis and inflammation [28]. The optimal treatment approach for patients with interstitial lung disease (ILD) remains an ongoing research and clinical investigation area. For the induction of remission, prednisone is favored, usually in combination with steroid-sparing agents. Pulse intravenous cyclophosphamide (IVCY) is a treatment option that has been reported to induce initial remission in patients with certain forms of interstitial lung disease (ILD), although non-complete resolution of pulmonary infiltrates with residual bibasilar linear opacities is commonly observed. However, complete resolution of pulmonary infiltrates may not always occur, and residual bibasilar linear opacities are commonly observed on imaging studies, as shown in Figure 1 [36,37].

### 4.3. Adult Respiratory Distress Syndrome (ARDS)

ARDS (acute respiratory distress syndrome) is a critical illness and a potentially life-threatening condition characterized by acute lung injury resulting in refractory hypoxemia despite supplemental oxygen treatment. It typically manifests as bilateral pulmonary infiltrates in imaging studies, which can be patchy or asymmetrical and may be accompanied by pleural effusions [38]. To date, a limited number of cases involving patients with both ARDS and APS have been documented (only 27 patients). Twenty patients evolved into catastrophic APS (Asherson’s syndrome) [38,39,40,41,42]. Patients with systemic vasculitis, antiphospholipid syndrome (APS), catastrophic APS (also known as Asherson’s syndrome), and Goodpasture’s syndrome are at risk of developing hemoptysis due to various underlying mechanisms, including diffuse alveolar damage (DAD). This pulmonary hemorrhage can be severe and rapidly progress to acute respiratory distress syndrome [18,43]. Pulmonary thromboembolism (PTE), accompanied by deep vein thrombosis (DVT), is one of the most frequently reported manifestations of antiphospholipid syndrome (APS) [18]. Acute respiratory distress syndrome (ARDS) is characterized by diffuse injury to the lung’s capillary endothelial and epithelial surfaces, leading to the development of non-cardiogenic pulmonary edema and severe hypoxemia. Evaluating antiphospholipid antibodies is indeed important in patients with acute respiratory distress syndrome (ARDS), especially if there is suspicion of an underlying autoimmune condition such as antiphospholipid syndrome (APS). The diagnostic criteria are crucial for identifying and diagnosing ARDS. These criteria include bilateral infiltrates on chest radiograph, which is indicative of diffuse lung injury and is a key feature of ARDS; the absence of left atrial hypertension, as evidenced by a pulmonary artery wedge pressure (PAWP) of less than 18 mm Hg, which helps differentiate ARDS from cardiogenic pulmonary edema; and partial pressure of arterial oxygen to fraction of inspired oxygen (PaO2/FIO2) ratio of 300 mm Hg or less (in ALI) or of 200 mm Hg or less (in ARD) [38]. This indicates significant hypoxemia despite the administration of supplemental oxygen [38]. In the clinical setting, patients with acute respiratory distress syndrome (ARDS) often present with worsening dyspnea and hypoxemia, necessitating intubation and mechanical ventilation to maintain adequate oxygenation. Upon examination, crackles may be auscultated on pulmonary examination, reflecting the presence of fluid-filled alveoli and interstitial edema. Chest radiographs typically reveal bilateral infiltrates, indicative of pulmonary edema, which may appear localized or diffuse. In addition to chest radiographs, computed tomography (CT) scanning of the thorax can provide further insights into the underlying pathology. CT scans may reveal evidence of pulmonary embolism (PE), pleural effusions, basilar atelectasis (collapse of the lung’s air sacs), and diffuse ground-glass opacities [38]. The development of ARDS can have various triggers, including acute increases in hydrostatic pressure from occlusive pulmonary emboli or microvascular emboli, causing vascular endothelial damage. In response to these insults, there is an influx of neutrophils into the pulmonary vasculature and alveolar spaces, leading to the release of pro-inflammatory cytokines and the activation of inflammatory cascades [37].

### 4.4. Pulmonary Arterial Hypertension (PAH)

Lupus pulmonary involvement may also evolve into a syndrome of pulmonary hypertension that is similar to idiopathic pulmonary hypertension. In this syndrome, patients present with dyspnea and a normal chest radiograph. They are mildly hypoxic and have a restrictive pattern on pulmonary function testing. Carbon dioxide diffusion capacity is reduced, and Raynaud’s phenomenon is frequently present. Doppler studies and cardiac catheterization confirm pulmonary hypertension. The prognosis is generally severe [14].

Pulmonary arterial vascular disease and associated pulmonary hypertension represent significant challenges in the management of scleroderma. The pulmonary vascular involvement in scleroderma can often progress silently, without obvious clinical symptoms, until it reaches an advanced stage. This indolent progression means that pulmonary hypertension may remain clinically undetectable until significant irreversible damage has occurred, leading to severe pulmonary hypertension and signs of right-sided heart failure. Significant pulmonary hypertension presents clinically with dyspnea on exertion and fatigue [30].

Transthoracic Doppler echocardiography (TTE) is a primary non-invasive screening tool for pulmonary arterial hypertension (PAH). One of its key functions is estimating right ventricular systolic pressure (RVSP), which indirectly reflects the pressure within the pulmonary arteries. Echocardiography is also crucial for evaluating other potential causes of pulmonary hypertension (PH), such as left heart valvular diseases (e.g., mitral valve stenosis) and myocardial diseases (e.g., heart failure with preserved ejection fraction). In these cases, TTE can identify abnormalities in the structure and function of the left heart that contribute to pulmonary venous hypertension, a distinct form of PH caused by elevated pressure in the pulmonary veins and capillaries due to left heart dysfunction [44].

Antiphospholipid syndrome (APS) is associated with various complications, including pulmonary hypertension (PH), which can arise due to chronic pulmonary thromboembolism. While there are limited treatment options for pulmonary hypertension, pulmonary thromboendarterectomy (PTE) has emerged as a viable treatment for a specific subset of patients with chronic thromboembolic pulmonary hypertension (CTEPH) [45].

### 4.5. Muscular Involvement

Respiratory muscle dysfunction, including both inspiratory and expiratory muscle involvement, has been documented in a significant portion of patients with dermatomyositis (DM) [46]; however, respiratory complications in dermatomyositis (DM) encompass a range of factors, including abnormal central respiratory drive, pharyngeal muscle impairment, interstitial lung disease, pneumonia due to aspiration or therapy-related immunosuppression, and drug-induced lung disease. The analysis of 70 patients with DM and diffuse interstitial lung disease shed light on the varied presentations of this condition. Among these patients, initial symptoms manifested in different ways: musculoskeletal symptoms (such as myalgias, arthralgias, and weakness) were the primary presentation in 25 patients (36%), while pulmonary symptoms were predominant in 21 patients (30%). Additionally, a notable subset of patients experienced a combination of musculoskeletal and pulmonary symptoms, indicating the complex interplay between these manifestations in DM [46].

### 4.6. Infections Related to Immunosuppressive Treatment and Differential Diagnosis 

Infections represent a significant cause of intensive care unit (ICU) admissions in patients with systemic rheumatic disorders, accounting for more than 50% of cases. A comprehensive evaluation is crucial in patients with systemic rheumatic disorders presenting with symptoms suggestive of organ involvement or infection. Respiratory failure due to pulmonary involvement is the most frequent problem encountered in these patients; therefore, when faced with a patient presenting with fever, cough, shortness of breath, and chest radiograph findings of pulmonary infiltrates, distinguishing between infectious and non-infectious causes of the infiltrates is essential.

Differentiating lung infections based on the infectious agent is crucial for guiding appropriate treatment and improving patient outcomes. Community-acquired pneumonia (CAP) can be caused by atypical pathogens, including mycoplasma pneumoniae, chlamydia pneumoniae, various respiratory viruses, including influenza, parainfluenza, respiratory syncytial virus (RSV), and Epstein–Barr virus (EBV). These pathogens often present with similar clinical symptoms, making it challenging to diagnose the specific etiology based solely on clinical presentation. The etiological diagnosis is often difficult due to the absence of absolute and accepted gold-standard diagnostic methods [47].

In contrast, fungal pathogens, particularly in immunocompromised individuals, can also cause pneumonia and may present with clinical features similar to those of other infectious agents. Host factors, such as immunosuppression (e.g., HIV infection, solid organ transplantation, chemotherapy), epidemiologic exposures (e.g., exposure to endemic fungi), radiographic patterns (e.g., nodular infiltrates, cavitation), and the presence of non-resolving pneumonia, should raise suspicion for fungal pneumonia.

To establish the etiology of pneumonia caused by atypical pathogens or fungal organisms, a combination of clinical assessment, radiographic findings, laboratory testing, and microbiological studies is often necessary. This may include serological tests, polymerase chain reaction (PCR) assays, culture, antigen detection, and molecular testing of respiratory specimens [48].

Patients receiving immunosuppressive therapy or those with leukopenia are at increased risk of opportunistic infections. Therefore, a high index of suspicion for opportunistic pathogens should be maintained in such cases. Concurrent exacerbation or new onset of extrapulmonary manifestations, such as arthritis, skin rash, or cardiac lesions, may suggest underlying disease activity rather than infection. This observation can help guide the diagnostic approach and treatment decisions. Sputum samples should be collected for staining and culture to identify common pathogens causing respiratory infections. Additionally, specific tests to isolate organisms or detect their antigens in sputum, blood, and urine should be performed to identify potential opportunistic pathogens.

Empiric antibiotic therapy should be adopted while the results of laboratory tests are pending. If sputum cultures are negative or inconclusive, a high-resolution CT scan can provide detailed imaging of the lung parenchyma, helping to characterize the pulmonary infiltrates further. Bronchoscopy with bronchoalveolar lavage (BAL) allows for direct sampling of the lower respiratory tract, aiding in identifying pathogens or inflammatory markers. In cases where the diagnosis remains unclear or when granulomatous or interstitial lung diseases are suspected, transbronchial biopsy can provide histological evidence. However, caution should be exercised in patients with a bleeding tendency, and appropriate precautions should be taken. If bronchoscopic samples and transbronchial biopsy are inconclusive or if the patient’s condition does not improve, consideration should be given to open-lung biopsy [7,49]. This invasive procedure allows for a larger tissue sample and may provide a definitive diagnosis. Prompt recognition of disease exacerbation is crucial to prevent rapid progression to severe lung injury and extrapulmonary organ failure, such as renal or cardiovascular dysfunction. Close monitoring of clinical and radiological parameters is essential for early intervention. If a rheumatologic disorder is suspected but not previously diagnosed, appropriate autoantibody diagnostic tests should be performed promptly after admission. This can help confirm the underlying rheumatic disease and guide further management. In cases where infectious etiology is suspected, prompt initiation of specific antimicrobial therapy is essential to prevent complications such as sepsis and multiple organ dysfunction syndrome (MODS). Delays in appropriate treatment can lead to adverse outcomes [7,49]. In critically ill patients presenting with clinical syndromes such as hemoptysis, renal failure, altered mental status, gastrointestinal bleeding, or acute abdomen, an aggressive diagnostic approach is warranted to identify the underlying cause promptly. Elevated C reactive protein (CRP) levels indicate inflammation and can be seen in infectious and non-infectious disease exacerbations. However, it is non-specific and cannot differentiate between the two. Elevated procalcitonin levels suggest bacterial or fungal infections, while normal or mildly elevated levels may indicate viral infections or flares of disease activity in rheumatic disorders. However, interpretation should be done with clinical findings and other laboratory results [50].

Patients with SLE face a greater risk of infections compared to the general population. This elevated susceptibility stems from the disease process and the immunosuppressive medications used to manage it [51]. Infections are a common complication in SLE, affecting more than half of patients. They can significantly impact morbidity and mortality, often leading to ICU admissions. The clinical presentations of infection and disease exacerbation can overlap, making diagnosis and management complex [49]. However, it is essential to meticulously rule out infection in SLE patients, as treating lupus exacerbations with immunosuppressive therapy can worsen underlying infections, leading to severe consequences [14]. The underlying disease process of SLE and the immunosuppressive therapy used in its treatment significantly elevate the risk of infections. However, determining the exact contribution of each factor is challenging. SLE patients experience a higher viral infection rate than the general population. While this is noted, no evidence suggests that these viral infections are more aggressive, resistant to therapy, or have a more chronic course than the general population [49]. Staphylococcus aureus, enterobacteriaceae, and non-fermentative gram-negative organisms are frequently implicated as causes of infections in SLE patients. SLE patients are predisposed to pneumococcal infections due to low complement levels, hyposplenism, opsonization, and chemotaxis deficit. Streptococcus pneumoniae, a common cause of community-acquired pneumonia, can lead to severe sepsis in SLE patients [52,53,54,55]. Patients with SLE who are treated with corticosteroids face a heightened risk of lung and brain diseases caused by Nocardia species [56]. Nocardiosis typically presents with lung nodules that may be cavitated, air-space consolidation, or pleural effusions. Chest wall extension can suggest the diagnosis.

Pulmonary involvement is a frequent complication in systemic lupus erythematosus (SLE), contributing to an overall mortality rate of 35%. Pneumocystis carinii infection is a significant concern, accounting for 12.5% of lethal infections in a classic series of opportunistic infections among SLE patients. Pneumocystis pneumonia primarily occurs in individuals receiving immunosuppressive medications, further emphasizing the importance of monitoring and preventing opportunistic infections in this population [57]. Despite predisposing factors such as immunosuppression, invasive fungal infections are less common than expected in SLE patients. Invasive aspergillosis, for instance, is uncommon but can occur in patients receiving high doses of corticosteroids. It often presents as nodular pulmonary lesions [14].

Patients affected by systemic lupus erythematosus (SLE), in particular those undergoing treatment with corticosteroids, residing in areas with a high prevalence of tuberculosis (TB), or exhibiting suggestive clinical histories or positive tuberculin skin tests, are at an elevated risk of developing tuberculosis. In patients with vasculitis, infections represent a significant cause of both morbidity and mortality [17]. This heightened susceptibility to infections is primarily attributed to the immunosuppressive therapies used to manage vasculitis, including cytotoxic agents and glucocorticoids. Glucocorticoids, such as prednisone, are particularly associated with an increased risk of infectious complications. Long-term treatment with doses exceeding 10 mg/die has been independently linked to a higher infection susceptibility. Pneumonia and sepsis are among the most common serious infections encountered in patients with systemic rheumatic diseases [58].

### 4.7. Other Diseases

#### 4.7.1. Lupus Pneumonitis

Acute lupus pneumonitis presents a diagnostic challenge as it shares symptoms with infectious pneumonia. Diagnosis involves excluding infections in patients with symptoms resembling contagious pneumonia. However, the reported incidence of acute lupus pneumonitis varies widely, from 0.9% to 11.7% [59], making it challenging to determine its exact frequency due to the limited data in the literature, primarily consisting of case reports and small case series. Common symptoms include dyspnea, cough, fever, and occasionally hemoptysis [59]. Chest radiographs typically reveal unilateral or bilateral alveolar infiltrates, although rare cases with normal radiographs have been reported. Pleural involvement is also frequently observed clinically and radiographically.

In some cases, lupus pneumonitis can be the initial presentation of systemic lupus erythematosus (SLE), occurring in approximately half of patients in one series. The mortality rate associated with lupus pneumonitis can be alarmingly high, reaching up to 50%. Therefore, promptly diagnosing and initiating appropriate therapy early in the disease are crucial for improving outcomes [59]. Given the severity of lupus pneumonitis and its potential to be life-threatening, healthcare providers should maintain a high index of suspicion, especially when encountering a young female patient with unexplained pulmonary infiltrates.

In the diagnostic workup of lupus pneumonitis, it is crucial to rule out other conditions that can mimic its clinical and radiographic features. This often involves obtaining blood and sputum cultures, and in some cases, more invasive procedures like bronchoscopy or open-lung biopsy may be necessary [60]. Pathological findings in lupus pneumonitis lack specificity [61], but certain features can be observed, including hematoxylin-eosin bodies or lupus erythematosus cells in rare instances [62]. Common histopathological findings include inflammation, tissue injury, alveolitis, alveolar necrosis, hemorrhage, edema, interstitial pneumonitis, hyaline membranes, interstitial pneumonitis, capillary thrombosis, and deposition of immunoglobulin and complement. Treatment typically involves high-dose corticosteroids, equivalent to prednisone, at 1–2 mg/kg/day, often leading to favorable responses [63]. However, in cases where patients do not respond adequately to corticosteroids, adjunctive agents may be necessary. Azathioprine is one of the most studied adjunctive therapies, although pulse cyclophosphamide, methotrexate, and plasmapheresis may also be used, sometimes in combination, to achieve better outcomes. Differential diagnosis between lupus pneumonitis and infections is possible after the determination of an increase of titers of anti-DNA with a decrease of C3/C4 and normal levels of C reactive protein and leukopenia, which are all typical of active SLE as well as normal levels of procalcitonin with a high level of erythrocyte sedimentation rate (ESR).

#### 4.7.2. Shrinking Lung Syndrome (SLS)

Shrinking lung syndrome (SLS) is a rare but recognized complication in patients with systemic lupus erythematosus (SLE) [64] and with other autoimmune diseases, as some case reports have described (one patient with Sjogren’s syndrome, one patient with rheumatoid arthritis, one patient with undifferentiated connective tissue disease and one patient with systemic sclerosis) [2]. Patients with shrinking lung syndrome (SLS) typically present with dyspnea, which can worsen when lying flat due to orthopnea, along with respiratory muscle dysfunction. Characteristic chest radiographic findings include small lung volumes, an elevated hemidiaphragm, and basilar atelectasis. Notably, these findings occur without significant pulmonary parenchymal or pulmonary vascular involvement, distinguishing SLS from other respiratory conditions [14]. Diaphragmatic dysfunction is a notable feature in conditions like shrinking lung syndrome, characterized by the progressive reduction in lung volume. Muscle weakness due to long-term corticosteroid use is unlikely to be the cause of muscle dysfunction in these patients and this is supported by the fact that shrinking lung syndrome has been documented in patients before they initiate steroid therapy, and corticosteroids have been shown to lead to clinical improvement in some cases.

Treatment response in shrinking lung syndrome can vary among individuals. While some patients may stabilize over time despite experiencing significant dyspnea and muscle weakness, others may require ongoing management and support [14].

#### 4.7.3. Laryngeal Stenosis

Subglottic stenosis poses a significant risk to patients with Wegener’s granulomatosis, affecting around 20% of individuals with this condition. Subglottic stenosis can present with a range of respiratory symptoms, including hoarseness, cough, dyspnea (difficulty breathing), and stridor (a high-pitched sound heard during breathing). The obstruction in the airway can result not only from the narrow lesion that occurs in the subglottic region itself [65] but also from trapped secretions in the tracheobronchial region at the level of stenosis [66]. Diagnosing subglottic stenosis can be challenging initially, as its symptoms may overlap with other respiratory conditions such as asthma or pulmonary diseases. Therefore, it is essential for patients presenting with these symptoms to undergo immediate evaluation by laryngoscopy, which allows direct visualization of the airway structures.

Regarding treatment, intratracheal dilatation combined with intralesional injections of glucocorticosteroids is an effective approach for managing subglottic stenosis [67].

#### 4.7.4. COVID-19 and Acute Respiratory Failure in Autoimmune Rheumatic Diseases

The advent of COVID-19, arising from the novel coronavirus SARS-CoV-2, presents formidable challenges for individuals with autoimmune rheumatic diseases, predisposing them to heightened risks of complications such as acute respiratory failure. Autoimmune rheumatic conditions, encompassing disorders like rheumatoid arthritis, lupus, and vasculitis, are characterized by an aberrant immune response, often resulting in inflammatory processes and organ damage, particularly affecting pulmonary function [68,69]. When compounded with the respiratory distress precipitated by COVID-19, the susceptibility to acute respiratory failure escalates [70,71]. The progression towards a severe clinical presentation is underscored by the intricate phenomenon termed a “cytokine storm”, activated by conceivable immunological mechanisms triggered by the viral incursion [71,72]. Perturbations in immune modulation, alongside potential autoinflammatory and autoimmune cascades, engender an exacerbated release of cytokines from immune cells, thereby exacerbating the inflammatory milieu [69,73]. Comprehensive management strategies necessitate vigilant monitoring, early symptom detection, and a collaborative approach involving rheumatologists and pulmonologists to optimize therapeutic interventions and mitigate the risk of severe respiratory sequelae.

#### 4.7.5. Secondary Diagnosis of Rheumatic Diseases in ICU

Secondary diagnoses of rheumatic diseases in the Intensive Care Unit (ICU) are not uncommon. Patients admitted to the ICU for various reasons may develop or exhibit symptoms of rheumatic diseases during their stay. While clinical signs can provide valuable clues for diagnosing autoimmune diseases, confirmation by rheumatologists with specialized expertise is often necessary for accurate diagnosis and management.

Considering the clinical signs, patients with suspected rheumatologic disease pathology admitted to the ICU may present arthritis or arthralgia, Raynauds’ phenomenon or digital ulcers, puffy hands and skin sclerosis, Gottrons’ papules, heliotrope rash, telangiectasias, calcinosis, dysphagia or fever of unknown origin.

In addition to these symptoms and signs, general laboratory exams, autoantibody exams, autoimmunity exams, and instrumental exams can identify patients with rheumatologic disease. Elevated levels of ESR and CRP are nonspecific markers of inflammation and can be seen in various conditions, including infectious and autoimmune diseases. However, they are commonly elevated in autoimmune inflammation, particularly in RA, where they are included as diagnostic criteria. Nonetheless, they lack specificity and should be interpreted with clinical findings. Reduction in the levels of complement proteins C3 and C4 can indicate active SLE and ANCA-associated vasculitis. These markers are included in the classification criteria for SLE [74]. Procalcitonin levels can help differentiate between infectious and non-infectious causes of inflammation. Elevated levels are more commonly associated with bacterial infections, whereas autoimmune diseases typically do not significantly affect procalcitonin levels. These laboratory markers serve as adjuncts to clinical evaluation and aid in diagnosing and monitoring autoimmune and inflammatory conditions [74].

Effective collaboration between rheumatologists and pulmonologists adept in managing ICU patients is indispensable for achieving an accurate differential diagnosis and delivering comprehensive care to individuals afflicted with autoimmune conditions such as systemic sclerosis (SSc), rheumatoid arthritis (RA), and systemic lupus erythematosus (SLE).

## 5. Discussion

Recent studies emphasize the critical importance of promptly identifying acute respiratory failure (ARF) as both a primary manifestation and potential complication of systemic rheumatic diseases. Collaborative efforts between ICU physicians and rheumatic specialists are crucial for providing comprehensive care to patients with systemic rheumatic diseases who require ICU admission. These conditions, including rheumatoid arthritis, systemic lupus erythematosus, scleroderma, and systemic vasculitis, can present with diverse clinical manifestations that may necessitate intensive care management. ANCA-associated vasculitis, particularly with severe pulmonary involvement, demands immediate attention despite diagnostic challenges amidst respiratory distress. Timely initiation of appropriate therapeutic interventions is crucial for improving prognosis, as delays in distinguishing disease exacerbations from infections can lead to adverse outcomes. Swift diagnosis and treatment are paramount to prevent organ system failures and adverse clinical consequences.

Observed mortality disparities among ICU-admitted patients with rheumatic diseases highlight the urgency of optimizing therapeutic strategies to reduce mortality rates. Incorporating multidisciplinary collaboration and targeted educational initiatives into clinical practice can enhance ARF management. Future research should focus on refining diagnostic algorithms and tailoring therapeutic interventions to improve clinical outcomes.

## 6. Conclusions

Acute respiratory failure can occur as the initial presentation or a complication of systemic rheumatic diseases or immunosuppressive therapy, like severe infections or drug reactions. Intensive care physicians must be aware of potential airway involvement in common rheumatologic disorders to manage challenges effectively during airway management and ICU admissions. Collaboration with rheumatic specialists is vital for accurate diagnosis and immediate treatment, especially for conditions such as rheumatoid arthritis (RA), systemic lupus erythematosus (SLE), and scleroderma, often leading to ICU admissions [7,22]. Systemic vasculitis, notably ANCA-associated vasculitis, may present with severe pulmonary involvement requiring ICU care. Early diagnosis and aggressive treatment are critical, given the potentially reversible nature of these diseases [23].

According to the literature, using laboratory markers such as PCR or neutrophil-to-lymphocyte ratio is not considered a consistent method to define or predict severe conditions or identify patients with a worse clinical outcome. In addition, elevated PCR levels cannot be used to distinguish infectious from non-infectious inflammation. For these reasons, it would be important to analyze and identify widely available laboratory markers that would help doctors in the differential diagnosis and identification of patients most at risk of having a worse clinical course of the disease.

Mortality rates in ICU-admitted rheumatic disease patients are notably higher than in other conditions, underscoring the urgency of tailored management. Differentiating between disease exacerbation and infection is crucial for timely therapy initiation and improved outcomes. Delayed treatment initiation can worsen organ dysfunction and escalate mortality rates, emphasizing the need for proactive management. Timely recognition and managing disease flares can mitigate complications and improve patient prognosis in ICU settings [1,24].

## Figures and Tables

**Table 1:** Autoimmune rheumatic disease in ICU: review of literature.

Author (Ref.)	Patients	Most Common Diagnosis	Mortality in ICU
Kollef et al. [11] 1992	36	RA* (67%), SLE* (22%), SCL* (14%), SS* (8%)	31%
Godeau et al. [8] 1992	69	69 RA (27%), vasculitides (27%), SLE (23%)	33%
Bouachour et al. [6] 1996	88	RA (35%), vasculitides (14%), SLE (14%), DMP* (5%)	37%
Godeau et al. [8] 1997	181	RA (33%), vasculitides (21%), SLE (20%), SCL (7%), DMP (7%)	33%
Pourrat et al. [4] 2000	33	RA (27%), SLE(24%), vasculitides (24%)	30%
Nguyen-Oghalai [12] 2000	52	RA (35%), SCL (20%), SLE (10%)	30%
Thong et al. [3] 2001	28	SLE (71%)	54%

RA*: Rheumatoid arthritis. SLE*: Systemic lupus erythematosus. SCL*: scleroderma. SS*: Sjogren syndrome. DMP*: polymyositis and dermatomyositis.

**Table 2:** Differential diagnosis of pulmonary hemorrhage syndromes [13].

Capillaritis WG*, CSS*, MPA*, idiopathic pauci-immune GN*, idiopathic pulmonary capillaritis, Goodpasture syndrome, Henoch–Schonlein purpura, SLE*, rheumatoid arthritis, polymyositis/dermatomyositis, scleroderma, mixed connective tissue disease, primary antiphospholipid antibody syndrome, essential cryoglobulinemia, Behcet disease, bone marrow transplantation, drug-induced disease (chemotherapeutic agents, diphenylhydantoin, propylthiouracil)
Bland hemorrhage: idiopathic pulmonary hemosiderosis, coagulopathy, mitral stenosis, inhalation injury, drug-associated disease (chemotherapeutic agents, penicillamine, trimellitic anhydride, amiodarone, nitrofurantoin)

WG*: Wegener’s granulomatosis. CSS*: cytokine storm syndormes. MPA*: microscopic polyangiitis. GN*: glomerulonephritis. SLE*: systemic lupus erythematosus.

**Table 3:** Differential diagnosis of pulmonary renal syndromes.

	SLE*	MPA*	WG*	Goodpasture
ANA*	++	--	--	--
anti-GBM*	--	+/-	+/-	++
anti-DNA*	++	--	--	--
ANCA*	--	++ (60% p-ANCA*, 30% c-ANCA*)	++ (80% c-ANCA*, 15% p-ANCA*)	+/+
C3/C4	↓↓	=	=	=

ANA*: antinuclear antibody. anti-GBM*: anti-glomerular basement membrane antibody. anti-DNA*: anti-deoxyribonucleic acid antibody. SLE*: systemic lupus erythematosus. MPA*: microscopic polyangiitis. WG*: Wegener’s granulomatosis. p-ANCA*: perinuclear anti-neutrophil cytoplasmic antibodies. ANCA*: antineutrophil cytoplasmic antibodies. +: laboratory exams present in the pathology. -: laboratory exams not present in the pathology. =: laboratory exams unchanged compared to normal conditions. ↓: very low laboratory exams in the pathology considered.

## Data Availability

Not applicable.
